# Obituary

**Published:** 1916-05

**Authors:** 


### OBITUARY

JAMES WARD HOUSE, D.D.S. Dr. House was born at Orangeville, a small town near Hamilton, Ontario, Canada, on December 5, 1864. He received a high school education and for a time was bookkeeper for a plumbing firm in Hamilton. Later he was copyist in the Court of Records. During this period he was a student of music and became proficient as a pianist. For a long time he had desired to enter one of the professions and saved his earnings to that end. He selected dentistry, and went to Chicago, graduating later from the Chicago College of Dental Surgery in 1886.

An acquaintance of his youth was practicing medicine in Grand Rapids, Michigan. This friend induced Dr. House to locate with him for the practice of his profession. They occupied the same suite of offices for a time. Later Dr. House joined Dr. W. A. Dorland in a suite of offices in the Widdicomb Building. He was married June 6, 1900, to Helen Louise Shepard, of Grand Rapids, who is now living at Pasadena, California.

After a few years he opened a suite of offices with Dr. Louis A. Roller, in the Gilbert Building, where he continued the practice of his profession until his health failed, in 1909. In March of 1910 he left for the West, remaining for a time in Pasadena. Not finding the benefit which he had hoped he went to San Diego, where he associated himself with a real estate firm, with which firm he remained until his death on April 8, 1916.

For some time before he left for the West he had suffered from rheumatic troubles. It finally settled into a severe case of arthritis. After living in San Diego for a time he seemed much improved, then the old trouble returned. His death was due, however, to excessive blood pressure and Bright's Disease, according to his attending physician, Dr. Robert H. Donnell.

He was a prominent member of the Masonic Fraternity and of many social organizations in Grand Rapids.

Dr. House was a man of unusual mental capacity and a most skillful dentist, and he enjoyed a large practice while in Grand Rapids. He took an active part in the dental society of his city and of the state, holding all the offices in these societies from time to time. His genial disposition and warm friendship made him a general favorite among his acquaintances. He was outspoken, frank and thoroughly true at heart. He was ever ready to sacrifice his own interests and pleasure for a friend. He often quoted the lines:

> "The man worth while is the man who can smile,
> When everything goes dead wrong."

He was truly a man worth while, and he put into practice, up to the time of his death, the sentiment expressed in this quotation. Probably no man was more active or more interested in his profession, and no one was more generally missed when he became so incapacitated that it became necessary for him to leave the state. Those who knew him in the time of his active work in Michigan's professional affairs will recall many instances of personal sacrifice made by him for his profession. We all recall his genial personality, and we can scarcely realize that he has gone. His many kind acts, and his professional attitude strongly attracted professional men to him, and sincere regrets will be expressed by a host of friends because he has passed from us forever.

